# PRKAR2B plays an oncogenic role in the castration-resistant prostate cancer

**DOI:** 10.18632/oncotarget.14044

**Published:** 2016-12-20

**Authors:** Jianjun Sha, Wei Xue, Baijun Dong, Jiahua Pan, Xiaorong WU, Dong Li, Dongming Liu, Yiran Huang

**Affiliations:** ^1^ Department of Urology, Ren Ji Hospital, School of Medicine, Shanghai Jiaotong University, Shanghai, China; ^2^ School of Biomedical Engineering, Shanghai Jiaotong University, Shanghai, People's Republic of China

**Keywords:** PRKAR2B, castration resistant, prostate cancer, CRPC, cell cycle

## Abstract

Castration-resistant prostate cancer (CRPC) is an advanced form of prostate cancer. Despite some progresses have been made, the mechanism of CRPC development is still largely unknown, including the genes involved in its development have not been well defined. Here, we identified*PRKAR2B* to be a gene over-expressingin castration-resistant prostate cancer by analyzing the different online databases. Followed functional validation experiments showed that PRKAR2B promoted CRPC cell proliferation and invasion, and inhibited CRPC cell apoptosis. Whole genome transcriptome and GO enrichment analyses of the knock-down of *PRKAR2B* in CRPC cells showed that PRKAR2B mainly accelerated cell cycle biological process and modulated multiple cell cycle genes, such as *CCNB1*, *MCM2*, *PLK1* and *AURKB*. Our study firstly identified *PRKAR2B* as a novel oncogenic gene involved in CRPC development and suggested it is a promising target for the future investigation and the treatment of CRPC.

## INTRODUCTION

At the early developmental stage, prostate cancer usually requires a constant supply of androgens for the development. However, metastatic prostate cancer flourishes despite the lack of androgens in the bloodstream, creating castration-resistant prostate cancer (CRPC). CPRC is an advanced form of prostate cancer characterized by disease progression following surgical or pharmaceutical castration (androgen deprivation). Compared with castration-sensitive prostate cancer patients, the survival rate of CRPC patients is low and its treat mentsarealso urgently needed to be improved [1].Therefore, it's very important to understand the mechanism of its development, such as the role of the critical genes regulating CRPC, to identify the effective therapeutic and diagnosis targets for the treatment of CRPC.

Gene expression and copy number profiling studies have identified recurrent gene fusions, chromosomal gains and losses, and deregulated pathways that drive prostate cancer progression to metastatic and lethal CRPC. These genomic changes include ETS gene family fusions, PTEN loss and androgen receptor (AR) amplification [2, 3]. Mutations of ETS gene family member, CHD1 and ETS2, were found in CRPC [3]. Recurrent mutationsin multiple chromatin- and histone-modifying genes, such as MLL2, were also identified in CRPC [3]. Mutation of AR collaborating factor FOXA1 repressed androgen signaling and increased tumor growth in CRPC. Other proteins, including ERG gene fusion product UTX (also known as KDM6A) and ASXL1, were mutated in CRPC and physically interacted with androgen receptor [3]. The mutation of N367T in 3β-hydroxysteroid dehydrogenase type 1 (3βHSD1) was observed in CRPC tumors and could enhanceihydrotestosterone (DHT) production in CRPC [4]. Despite those progresses, more efforts are needed to identify more promising targets for the treatment of CRPC.

In order to identify the target genes, which play important roles in the development of CRPC, we screened GEO and other related databases and mined relevant literatures through bioinformatic analyses. We identified *PRKAR2B*, a gene encoding cAMP-dependent protein kinase type II-beta regulatory subunit, to be an important oncogenic gene in CPRC. Knock-down of *PRKAR2B* in CRPC cells increased cell apoptosis and inhibited cell proliferation and invasion. The downstream genes and pathways of *PRKAR2B* in CRPC cells were identified by the whole genome transcriptomic and GO enrichment analyses. Therefore, we concluded thatPRKAR2B seemed to be a novel oncogenic gene involved CRPC development.

## RESULTS

### Identify the potential gene over expressing in CRPC through screening online databases

**Figure 1:**
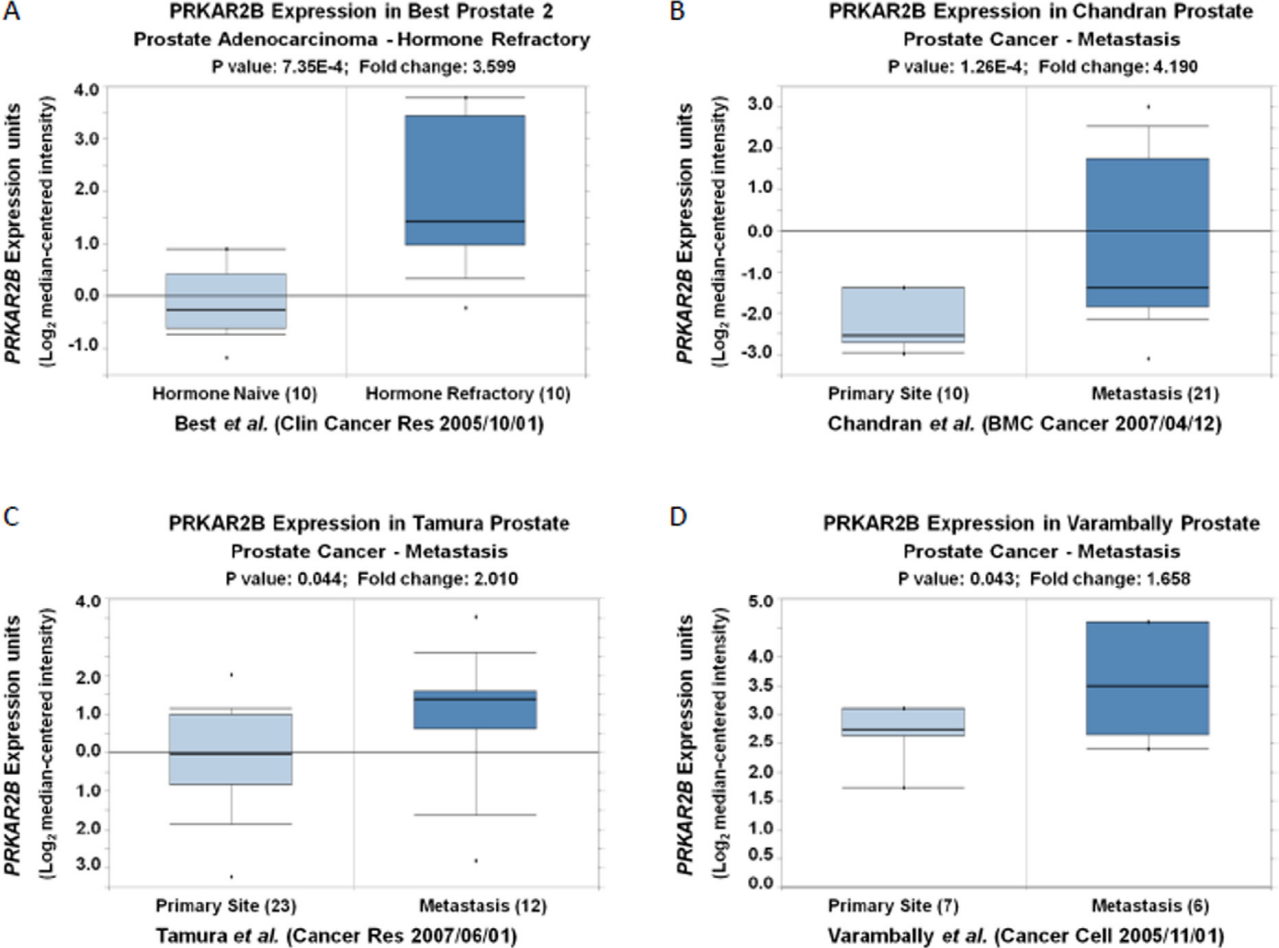
The mRNA expression of PRKAR2B is increased in the patients with hormone refractory and/or metastatic prostate tumors compared to that in controls (**A**) Oncomine analysis show that the expression of *PRKAR2B* (Log2 median-centered intensity) in hormone naïve prostate adenocarcinoma and hormone refractory prostate adenocarnoma (*P <* 0.001). (**B**–**D**) Oncomine analysis show that the expression of *PRKAR2B* (Log2 median-centered intensity) in prostate tumor tissue from primary site and metastatic site (*P <* 0.05).

In order to identify the key novel genes regulating the development of CRPC, we screened the GEO databases and analyzed one of representative databases (Table 1). The comparisons of differentially changed gene expression in prostate cancer were made among human prostate cancer xenograft models in the stages of androgen-dependent growth (AD samples), castration-induced regression nadir (ND samples) and castration-resistant growth (CR samples) (Table 1). We performed two comparisons. One was performed between ND and AD samples (Comparison 1) and the other was analyzed between CR and ND samples (Comparison 2). Table 2 showed the distribution of differentially expressed genes (DEGs) in Comparison 1 and Table 3 showed the distribution of DEGs in Comparison 2. We analyzed 22300 probes and 12222 genes in both comparisons. In those comparisons, we used this criterion (|log(fold change)| >1 and *p value* < 0.05) to define the differentially changed genes to be statistically significant. In Comparison 1(ND vs AD), we identified 377 out of 12222 genes had significant changes (Table 2). Among those genes, 63 genes were up-regulated and 314 genes were down-regulated (Table 2). Comparison 1 (ND vs AD) was to identify those genes contributing the androgen-dependent prostate cancer development. In contrast, in Comparison 2(CR vs ND), only 112 genes were significantly changed, among which 91 genes were up-regulated and 21 genes were down-regulated (Table 3). Comparison 2 (CR vs ND) was to identify those genes contributing the castration-resistant prostate cancer development. However, those changed genes identified in Comparison 2 also included the androgen-dependent genes which might be induced during the CRPC development. To exclude those genes, we did the overlapping analysis between Comparisons 1 and 2 to specifically identify those genes only changed in the CRPC. Those genes only changed in Comparison 2 were regarded to be more important in CRPC development. We summarized DEGs regulation status in Comparison 1 and Comparison 2 in Table 4. We identified 72 up-regulated genes and 18 down-regulated genes exclusively in Comparison 2 but not in Comparison 1 ((log2(fold change) > 1 & adjusted *p value* < 0.05) (Table 5 and Table 6). Among those specifically dysregulated genes in castration-resistant samples, EP4 (PTGER4) was listed in our Table 5 and it's a validated target for the treatment of castration-resistant prostate cancer [5]. By further combined Oncomine analysis of other database (Figure 1), we identified *PRKAR2B* as one of the potential target genes contributing to CRPC development. In detail, the expression of *PRKAR2B* was significantly increased in the prostate patients with hormone refractory (Figure 1A) [6], and elevated expression of *PRKAR2B* was also found in metastasized prostate tumors (Figure 1C–1D) compared to that in primary prostate tumors [7–9]. These evidences suggested that undefined function of *PRKAR2B* might play an important role in the development of CRPC.

**Table 1:** Comparisons of gene expression in prostate cancer were made among human prostate cancer xenograft in the stage of androgen-dependent growth (AD samples), castration-induced regression nadir (ND samples) and castration-resistant regrowth (CR samples) in GEO database

Title			
Organism	Homo sapiens		
GEO ID	GSE21887		
Sample Information			
Data Contents	Human prostate cancer xenograft in the stage of androgen-dependent growth (AD samples)	Human prostate cancer xenograft in the stage of castration-induced regression nadir (ND samples)	Human prostate cancer xenograft in the stage of castration-resistant regrowth (CR samples)
	GSM544229.CEL GSM544230.CEL GSM544231.CEL GSM544232.CEL	GSM544233.CEL GSM544234.CEL GSM544235.CEL GSM544236.CEL	GSM544237.CEL GSM544238.CEL GSM544239.CEL GSM544240.CEL
Platform	Affymetrix Human Genome U133 Plus 2.0 Array		

**Table 2:** Statistical distribution of DEGs in comparison 1 (The up or down level was obtained by comparing ND samples to AD samples)

	Probe		Gene	
All	22300		12222	
|log2(fold change)| > 1 && adjusted *p* value < 0.05	491	81 (up) 410 (down)	377	63 (up) 314 (down)

**Table 3:** Statistical distribution of DEGs in comparison 2 (The up or down level was obtained by comparing CR samples to ND samples)

	Probe		Gene	
All	22300		12222	
|log2(fold change)| > 1 && adjusted *p* value < 0.05	129	105 (up) 24 (down)	112	91 (up) 21 (down)

**Table 4:** DEGs regulation status in two comparisons (comparison 1 was between ND samples and AD samples, while comparison 2 was between CR samples and ND samples)

DEGs regulation status in comparison 1&2		Gene Count
Commonly changed	Commonly up-regulated	0
	Commonly down-regulated	0
Changed only in comparison 1	Up in comparison 1 while no change in comparison 2	60
	Down in comparison 1 while no change in comparison 2	295
Changed only in comparison 2	Up in comparison 2 while no change in comparison 1	72
	Down in comparison 2 while no change in comparison 1	18
Others	Up in comparison 1 while down in comparison 2	3
	Down in comparison 1 while up in comparison 2	19

**Table 5:** 72 up-regulated genes only changed in comparison 2 (log2(fold change) > 1 && adjusted *p* value < 0.05)

Gene Symbol	Log_2_(Fold Change)	*P* Value	Adjusted *P* Value	Gene Symbol	Log_2_(Fold Change)	*P* Value	Adjusted *P* Value
GUCY1A2	6.94	3.17E–10	3.79E–06	TSPAN5	1.67	3.87E–05	2.07E–02
LINC00844	6.03	1.25E–04	3.42E–02	APBB2	1.67	4.39E–05	2.22E–02
GRIN3A	5.65	1.68E–10	3.79E–06	HMGCS1	1.65	1.55E–04	3.57E–02
PTGER4	5.31	9.09E–05	2.99E–02	CASP4	1.62	5.73E–05	2.52E–02
LUM	4.63	1.75E–04	3.78E–02	C2orf76	1.53	2.27E–04	4.19E–02
NPR3	4.27	7.19E–05	2.73E–02	WWC3	1.5	1.06E–04	3.16E–02
STXBP6	3.88	6.79E–05	2.66E–02	AQP3	1.47	3.08E–04	4.82E–02
NR5A2	3.41	9.43E–07	2.86E–03	PARP9	1.46	7.36E–05	2.75E–02
GABRA1	3.33	2.36E–04	4.21E–02	PPAP2A	1.45	3.26E–04	4.87E–02
NUDT11	3.25	2.93E–06	5.01E–03	STX19	1.43	1.17E–05	1.09E–02
ADAM7	3.25	6.20E–07	2.12E–03	EIF4E3	1.42	1.19E–05	1.09E–02
SHISA9	3.08	1.20E–07	6.55E–04	ZFY	1.4	3.34E–04	4.87E–02
CCDC68	3.06	1.46E–07	6.63E–04	NEDD4L	1.39	3.47E–04	4.89E–02
PRR16	3	9.53E–05	3.04E–02	MTHFD2L	1.37	1.99E–05	1.47E–02
NLGN1	2.92	2.17E–04	4.18E–02	RIT1	1.3	3.55E–04	4.92E–02
PCDHB6	2.84	2.95E–04	4.71E–02	SRPK2	1.26	1.96E–04	3.87E–02
PRKAR2B	2.63	1.28E–05	1.09E–02	GABARAPL1	1.22	2.95E–04	4.71E–02
PPP1R3D	2.51	1.43E–04	3.51E–02	CEP192	1.19	1.80E–05	1.40E–02
ERO1LB	2.49	5.89E–06	8.94E–03	TLE4	1.19	1.43E–04	3.51E–02
KATNAL1	2.39	3.41E–04	4.87E–02	TMEM87A	1.18	3.64E–05	2.03E–02
SYTL4	2.36	1.81E–04	3.78E–02	SLC44A2	1.15	3.16E–04	4.85E–02
PKIB	2.34	8.16E–06	9.42E–03	ELF4	1.15	3.39E–04	4.87E–02
ZDHHC14	2.24	6.12E–05	2.65E–02	CDC42SE2	1.13	3.37E–04	4.87E–02
PTPRJ	2.23	4.49E–05	2.23E–02	OSTM1	1.11	3.09E–04	4.82E–02
RERG	2.18	1.45E–05	1.20E–02	KCTD16	1.09	1.23E–04	3.40E–02
FAM105A	2.06	1.67E–04	3.78E–02	PTPRK	1.08	1.31E–04	3.42E–02
ST8SIA1	2	2.92E–05	1.80E–02	BMPR2	1.08	9.59E–05	3.04E–02
COBLL1	1.94	1.46E–04	3.54E–02	JKAMP	1.07	3.28E–04	4.87E–02
MIA2	1.86	2.69E–04	4.51E–02	EIF1AY	1.07	3.40E–04	4.87E–02
FAM49A	1.82	7.42E–06	9.42E–03	FAM115A	1.06	3.13E–04	4.85E–02
ZNF697	1.81	2.60E–05	1.69E–02	CREM	1.06	1.81E–04	3.78E–02
ZNF124	1.79	2.08E–05	1.49E–02	JADE1	1.04	6.44E–05	2.66E–02
FBXO6	1.76	2.25E–04	4.19E–02	SAE1	1.04	3.41E–04	4.87E–02
GLYATL2	1.76	8.12E–05	2.91E–02	SHANK2	1.03	1.33E–04	3.42E–02
RND3	1.74	2.08E–04	4.06E–02	ZMIZ1	1.02	3.20E–04	4.87E–02
ZNF275	1.7	5.03E–05	2.37E–02	UPP1	1.01	1.26E–05	1.09E–02

**Table 6:** 18 down-regulated genes only changed in comparison (log2(fold change) < −1 && adjusted *p* value < 0.05)

Gene Symbol	Log_2_ (Fold Change)	*P* Value	Adjusted *P* Value
ACADVL	−1.10	3.52E–05	2.03E–02
ACSM3	−1.34	2.63E–04	4.49E–02
BEND4	−3.12	3.71E–04	5.00E–02
CGN	−1.45	2.25E–04	4.19E–02
DCUN1D1	−1.23	1.31E–04	3.42E–02
EFNB2	−3.63	2.20E–04	4.18E–02
FAM120A	−1.31	1.11E–04	3.22E–02
ISYNA1	−1.75	2.52E–05	1.68E–02
LSM12	−2.10	2.96E–05	1.80E–02
MAPT-AS1	−1.01	1.31E–04	3.42E–02
MTMR11	−1.26	1.28E–04	3.42E–02
NTM	−1.10	2.76E–04	4.56E–02
PON2	−1.13	2.56E–04	4.44E–02
RBM39	−1.02	8.87E–05	2.99E–02
SCUBE2	−4.38	1.13E–04	3.23E–02
TMEM184A	−1.56	4.87E–05	2.33E–02
WASL	−1.64	6.70E–05	2.66E–02
ZNF385B	−2.39	1.74E–04	3.78E–02

**Table 7:** GO pathways consisted of significantly and differentially changed cell cycle genes in DU145 cells after knock-down of PRKAR2B (p value < 0.01)

ID	Description	*P* value	Adjust *p* value
GO:0000278	mitotic cell cycle	1.15E-46	2.43E-43
GO:0007049	cell cycle	1.33E-46	2.43E-43
GO:1903047	mitotic cell cycle process	1.76E-40	2.14E-37
GO:0022402	cell cycle process	6.06E-40	5.53E-37
GO:0007067	mitotic nuclear division	1.67E-27	1.22E-24
GO:0000280	nuclear division	3.11E-27	1.89E-24
GO:0051301	cell division	5.13E-27	2.68E-24
GO:0048285	organelle fission	1.07E-26	4.87E-24
GO:0044763	single-organism cellular process	1.71E-26	6.95E-24
GO:0044770	cell cycle phase transition	2.96E-25	1.04E-22

### PRKAR2B promotes CRPC cell growth, invasion and survival

**Figure 2:**
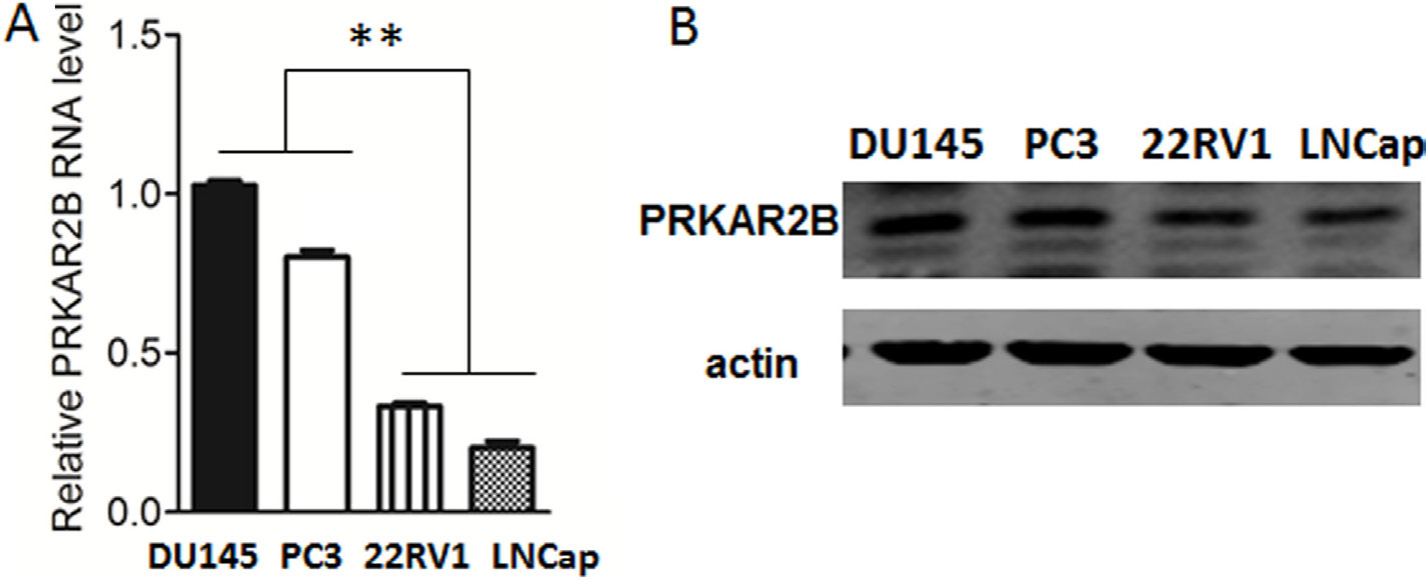
Expression of PRKAR2B in prostate cancer cell lines (**A**) The mRNA expression level of *PRKAR2B* in DU145, PC3, 22RV1 and LNCap cells was detected by Real-Time qPCR. (**B**) The protein expression level of PRKAR2B in DU145, PC3, 22RV1 and LNCap cells was detected by western-blot, and β-actin was the internal loading control (**P < 0.01).

To investigate the expression of PRKAR2B in prostate cancer cells, we examined PRKAR2B expression by real-time PCR for RNA expression and by western blot for protein expression in four prostate cancer cell lines,DU-145, PC-3,22RV1 and LNCaP. DU-145, PC-3 and 22RV1 are castration resistant prostate cancer cell lines and LNCaP is castration sensitive prostate cancer cell line which is sensitive to castration [10, 11]. Figure 2 showed that DU-145 and PC-3 has higher expression of PRKAR2B than22RV1 and LNCaP (Figure 2A and 2B). The castration sensitive prostate cancer cell line, LNCap, had the lowest expression of PRKAR2B among the four cell lines(Figure 2A and 2B).

**Figure 3:**
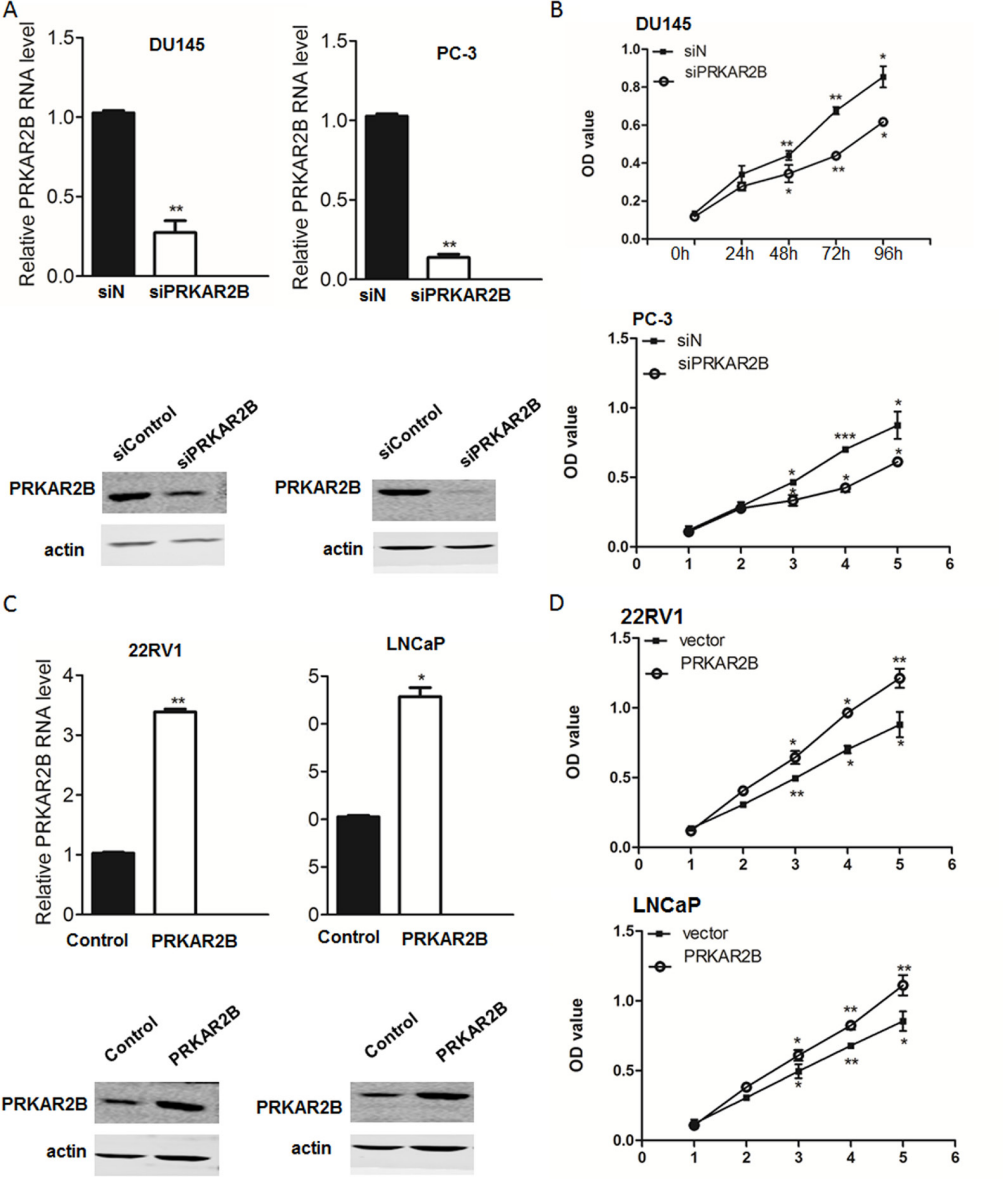
PRKAR2B promotes CRPC cell proliferation (**A**) The mRNA and protein expression level of PRKAR2B was examined by Real-Time qPCR (bar graph) and western blot, respectively, in DU145 and PC3 cells with or without PRKAR2B knockdown. (**B**) MTT assay shows cell viability of DU145 cells (upper panel) and PC3 cells (lower panel) withor without PRKAR2Bknockdown. OD value at 570nmwas tested at 0 h, 24 h, 48 h, 72 h and 96 h after siRNA transfection. siN: negative control siRNA;siPRKAR2B: PRKAR2B siRNA. (**C**) The mRNA and protein expression level of PRKAR2B was examined by Real-Time qPCR (bar graph) and western blot, respectively, in 22RV1 and LNCap cells after transfection of PRKAR2B plasmid for 48 hours. (**D**) MTT assay showed cell proliferation of 22RV1 cells(upper panel) and LNCap cells(lower panel) with or without PRKAR2B plasmid transfection. OD value at 570nmwas tested at 0 h, 24 h, 48 h, 72 h and 96 h after plasmid transfection (*P < 0.05; **P < 0.01; ***P < 0.001).

To investigate the function of PRKAR2B in these prostate cancer cell lines, we knock-downed PRKAR2B in DU-145and PC-3using siRNA and observed cell proliferations of both cell lines were inhibited by the knock-down of PRKAR2B (Figure 3A and 3B). Over expression of PRKAR2B in 22RV1 through PRKAR2B plasmid transfection promoted cell proliferation without androgen supplementation. Interestingly, over expression of PRKAR2B in LNCaP also increased cell proliferation but it's not significant as that in 22RV1 (Figure 3C and 3D). Meanwhile, knock-down of PRKAR2B expression in LNCaP did not significantly change cell proliferation though there's a decreased trend (Supplementary Figure S1B). These results suggested that PRKAR2B had more effect to regulate cell proliferation in castration-resistant prostate cancer cell compared to that in castration-sensitive prostate cancer cell.

**Figure 4:**
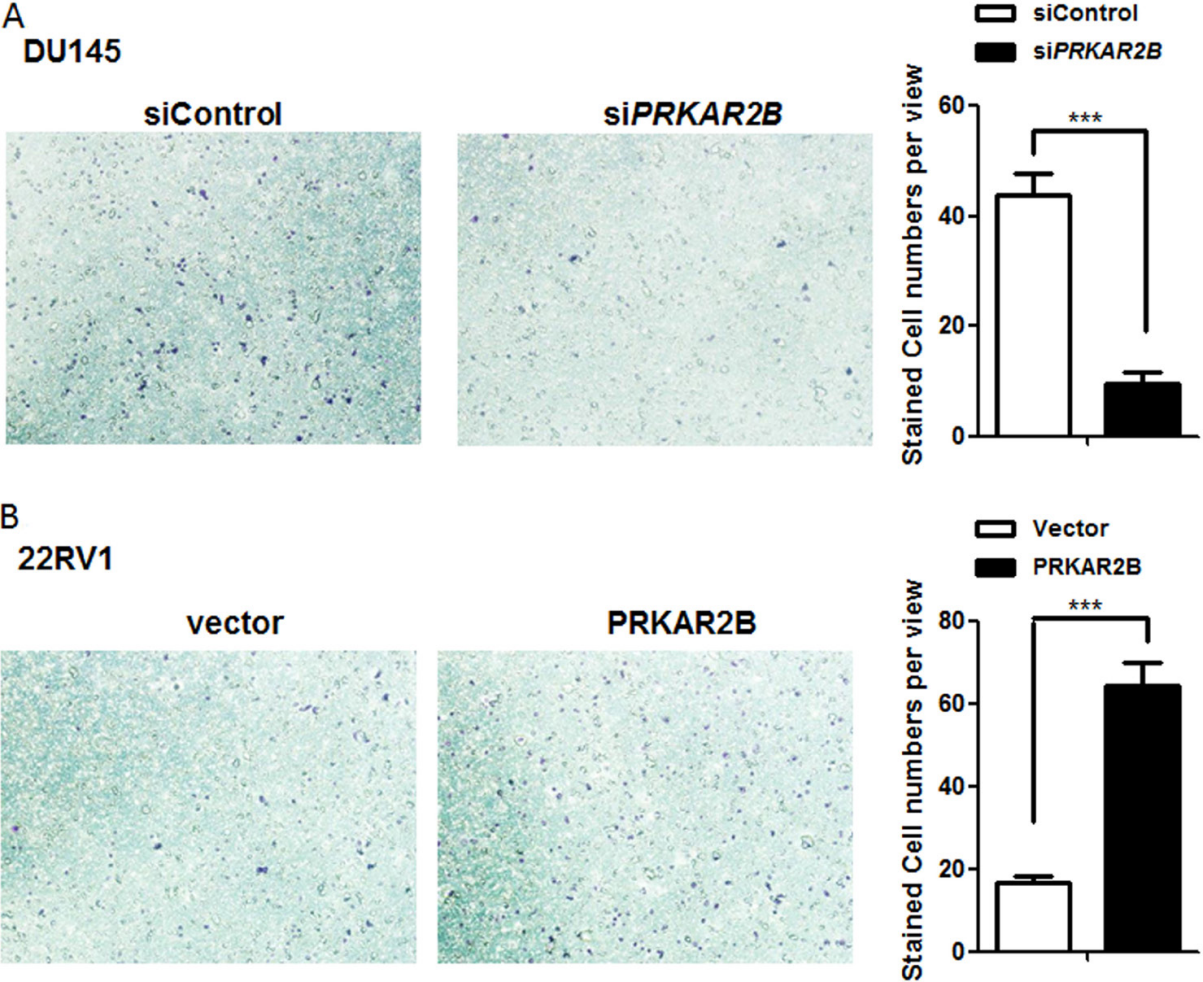
PRKAR2B promotes CRPC cell invasion (**A**) DU145 cell invasion ability was examined by invasion chamber assay after transfection of PRKAR2B siRNA for 48 hours. The bar graph shows the average number of the invaded cells per field. (**B**) Cell invasion ability of 22RV1 was examined by invasion chamber assay after transfection of PRKAR2B plasmid for 48 hours. The bar graph shows the average number of the invaded cells per field(***P < 0.001).

The function of PRKAR2Bin regulating cell invasion was also investigated in DU-145 and 22RV1 cells. Knock-down of PRKAR2B expression through siRNA in DU-145 cell impaired DU-145 cell invasion capability (Figure 4A) and over-expression of PRKAR2B through PRKAR2B plasmid transfection in 22RV1 enhanced cell invasion capability (Figure 4B). However, knock-down of PRKAR2B expression through siRNA in LNCaP did not significantly alter cell invasion capability (Supplementary Figure S1C), indicating that PRKAR2B plays more oncogenic roles, such as cell invasion, in castration-resistant prostate cancer cell compared to that in castration-sensitive prostate cancer cell.

**Figure 5:**
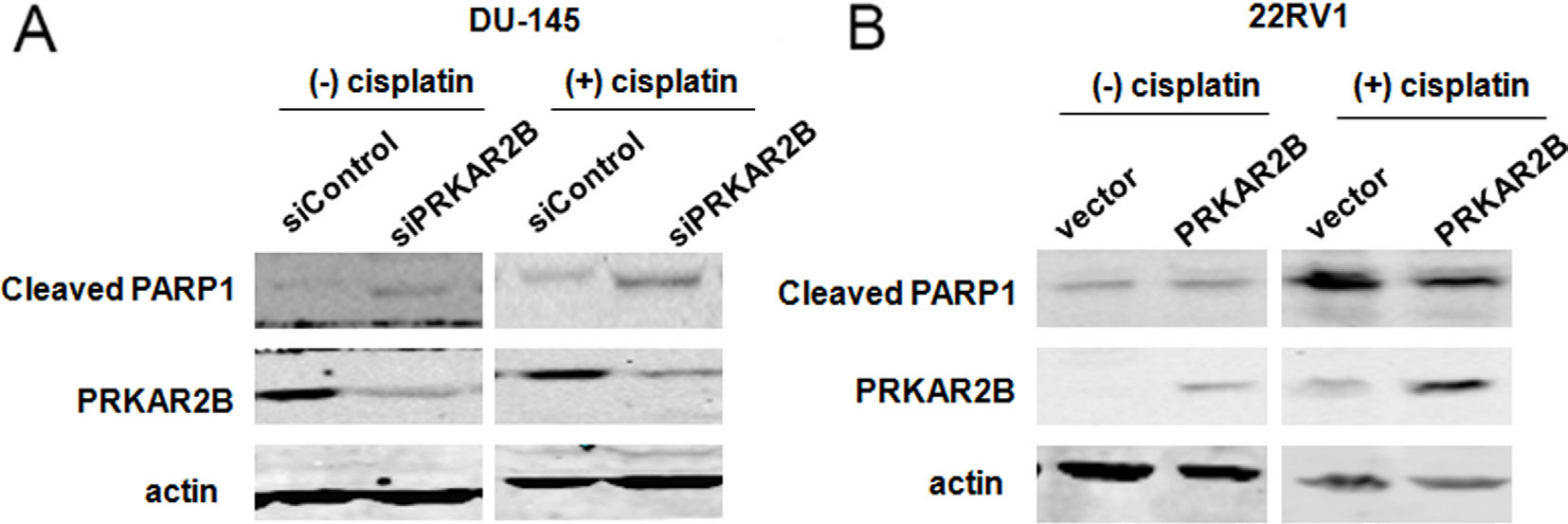
PRKAR2B plays the role of anti-apoptosis in CRPC cells (**A**) DU145 cell was transfected with PRKAR2B siRNA for 48 hours, and then treated with DMSO (left panel) or 10 μM cisplatin (right panel) for 24 hours. The protein expression of PRKAR2B and cleaved PARP1 were examined through western blot. Actin was the internal loading control. (**B**) 22RV1 cells was transfected with PRKAR2B plasmid for 48 hours, and then treated with 10 μM cisplatin for 24 hours. The protein expression of PRKAR2B and cleaved PARP1 were examined through western blot. Actin was the internal loading control.

Furthermore, knock-down of PRKAR2B in DU-145 cell using siRNA increased the expression of cleaved PARP (Figure 5A, left panel), a cell apoptosis marker, indicating PRKAR2B is required for prostate cancer cell survival and knockdown of PRKAR2B expression will induce cell apoptosis. Cisplatin is a common drug for the chemotherapy treatment of diverse cancers, including prostate cancer, and also can induce cell apoptosis [12–15]. We observed that knock-down of PRKAR2B expression in DU-145 sensitized the cell to cisplatin induced cell apoptosis, indicated by the increased PARP cleavage (Figure 5A, right panel), suggestingPRKAR2B cause the anti-apoptosis of CRPC cells under the treatment of cisplatin. In consistent with this results, over expression of PRKAR2B in 22RV1decreased cell apoptosis caused by cisplatin treatment (Figure 5B, right panel) though this effect was hard to observe without cisplatin (Figure 5B, left panel).

Taken together, PRKAR2B promotes cell proliferation, invasion and survival in castration-resistant prostate cancer cells.

### Whole genome transcriptomic analysis of the target genes of PRKAR2B in CRPC cells

**Figure 6:**
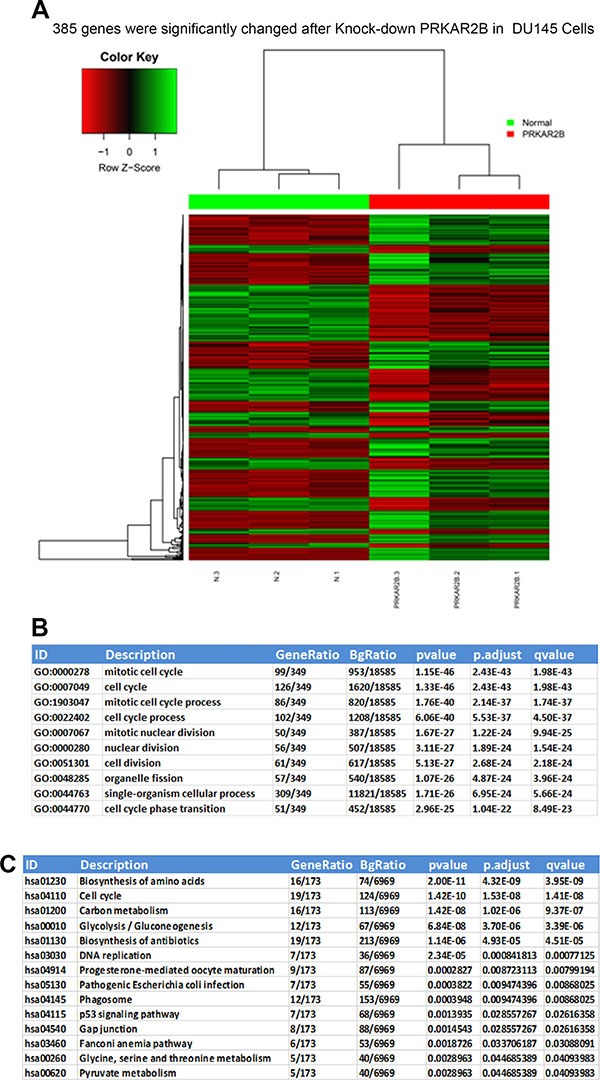
Whole genome transcriptome and pathway analyses of significantly and differentially changed genes regulated by PRKAR2B in DU145 cells (**A**) Heat-map of RNA-SEQ shows that385 genes were significantly changed in DU145 cells after knockdown of PRKAR2B expression. (**B** and **C**)The summary of top 10 changed biological processes or pathways after knock-down of PRKAR2B in DU145 cells by using GO biological process analysis (B) and KEGG pathway analysis (C).

To investigate the mechanism how PRKAR2B regulates CRPC, we knock-downed PRKAR2B in DU-145 cells, and then performed the RNA-SEQ to examine the whole genomic gene expression profile after inhibition of PRKAR2B. We identified 385 genes which were significantly changed after knock-down of PRKAR2B in DU145 cells (Figure 6A). GO biological process analysis indicated that those genes were significantly involved in mitotic cell cycle, cell cycle, mitotic cell cycle process, cell cycle process, mitotic nuclear division, nuclear division, cell division, organelle fission, single-organism cellular process and cell cycle phase transition (Figure 6B). KEGG pathway analysis also showed those genes mainly participated in regulation of cell cycle and DNA replication signaling pathways (Figure 6C). These results indicated there’sastrong correlation between PRKAR2B and cell cycle and cell proliferation processes.

### Identify key downstream genes involved in PRKAR2B signaling pathway

**Figure 7:**
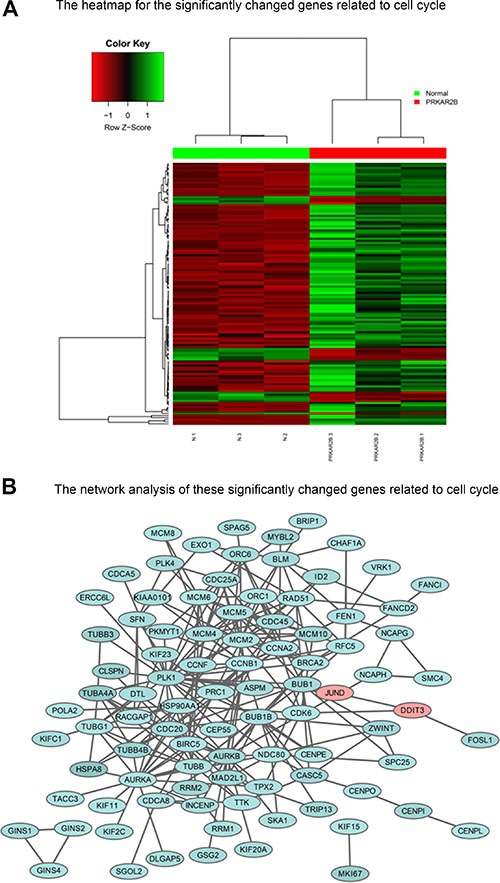
The cell cycle genes regulated by PRKAR2B in DU145 cells (**A**) Heat-map of the significantly and differentially changed cell cycle genes in DU145 cells after knock-down of PRKAR2B. (**B**) Network analysis of the significantly and differentially changed cell cycle genes DU145 cells after knock-down of PRKAR2B.

**Figure 8:**
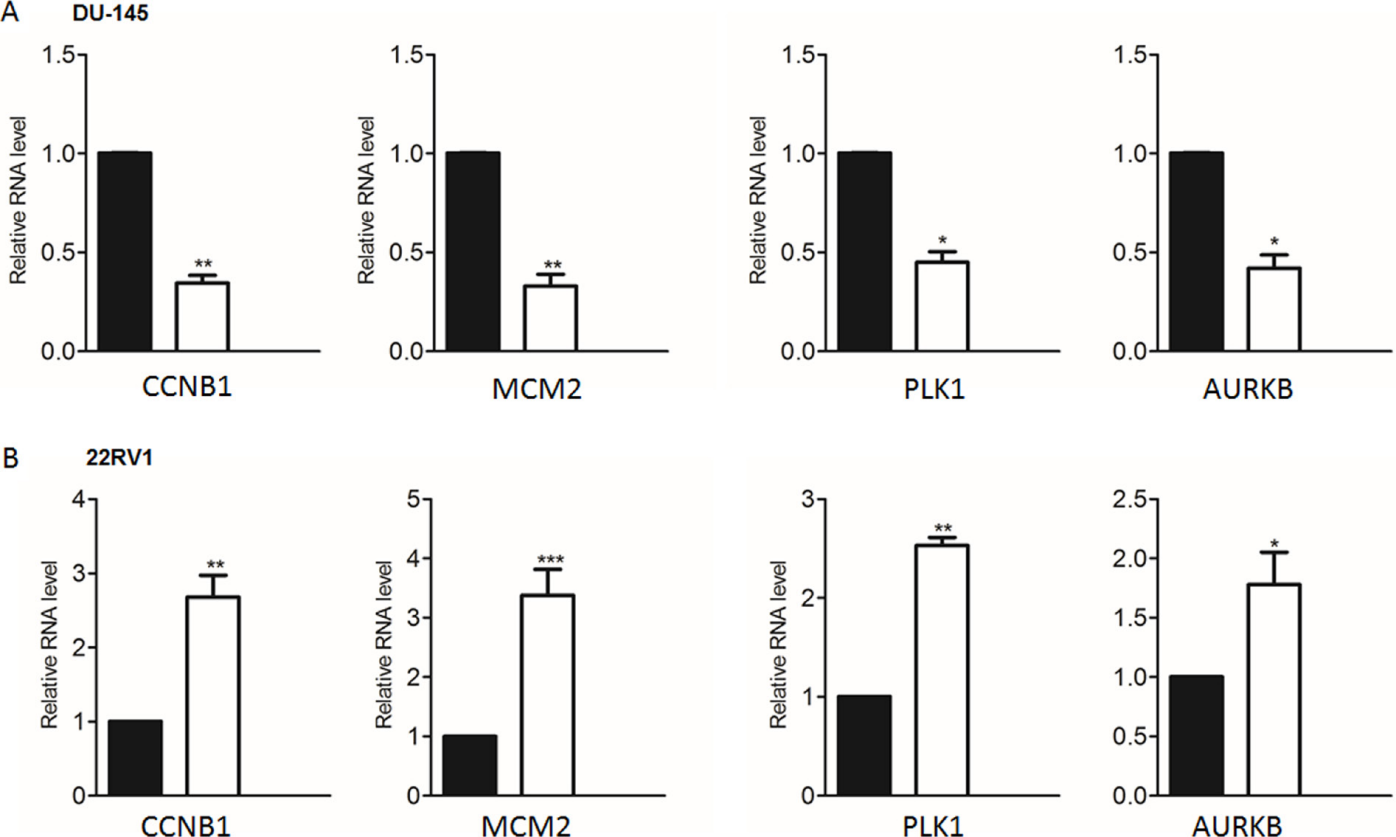
Validate the representative cell cycle genes regulated by PRKAR2B in castration-resistant prostate cancer cell lines (**A**) Real-Time qPCR was used to examine the expression levels of four cell cycle genes in DU145 cells after knock-down of PRKAR2B. (**B**) Real-Time qPCR was performed to examine the expression levels of four cell cycle genes in 22RV1 cells after overexpression of PRKAR2B by transfection of its plasmid (*P < 0.05; **P < 0.01; ***P < 0.001).

**Figure 9:**
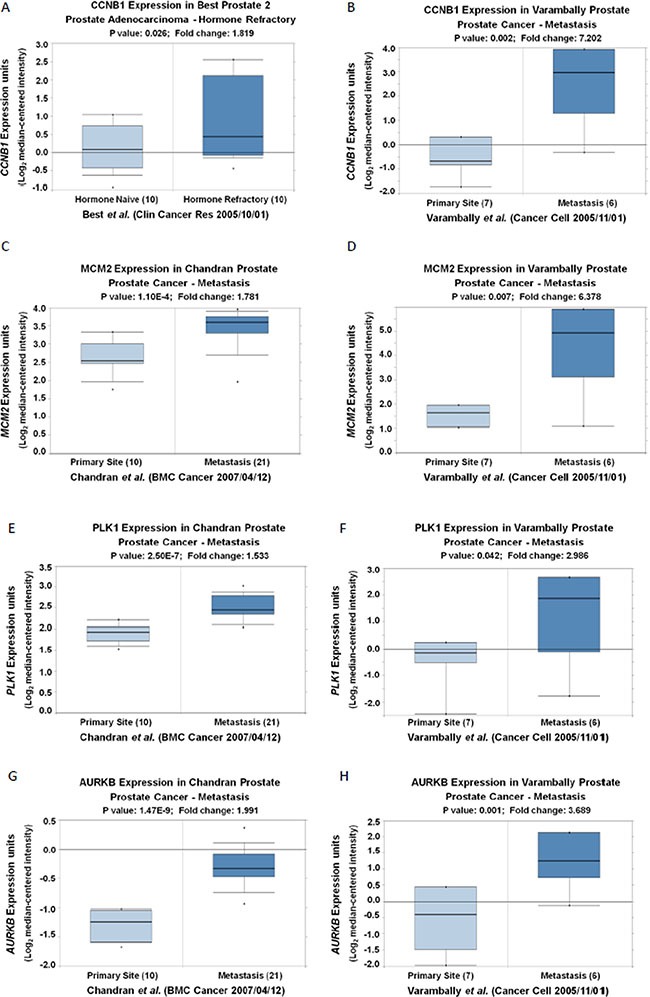
The mRNA expression levels of CCNB1, MCM2, PLK1 and AURKB are increased in patients with hormone refractory and/or metastatic prostate tumors compared to those in controls (**A**) Oncomine analysis show that the expression of *CCNB1* (Log2 median-centered intensity) in hormone naïve prostate adenocarcinoma and hormone refractory prostate adenocarnoma (*P <* 0.05). (**B**–**H**) Oncomine analysis show that the expression of *CCNB1* (B), *MCM2* (C and D), *PLK1* (E and F) and *AURKB* (G and H) (Log2 median-centered intensity) in prostate tumor tissues from primary site and metastatic site (*P <* 0.05).

To determine the key cell cycle genes regulated by PRKAR2B in CRPC cells, we further analyzed those significantly changed genes related to cell cycle in Figure 6B. We extracted those cell cycle genes (Figure 7A) and Table 7, and then performed the network analysis on these significantly changed genes (Figure 7B). We chose some hub genes, which had more connections to other genes in the whole network, such as *CCNB1MCM2*, *PLK1* and *AURKB*, to validate their mRNA expression changes after knock-down of *PRKAR2B*. In DU145 cells, the expression of those target genes, *CCNB1MCM2*, *PLK1* and *AURKB*, were decreased after knock-down of *PRKAR2B* usingsiRNA (Figure 8A). Meanwhile, over expression of PRKAR2B in 22RV1 cells led to the increased expressions of *CCNB1*, *MCM2*, *PLK1* and *AURKB* (Figure 8B). Notably, in the same prostate cancer patient databases as those in Figure 1, the higher mRNA expression of *CCNB1* was observed in the patients with hormone refractory compared to that in the patients having hormone naïve prostate cancer (Figure 9A), and the mRNA expression levels of *CCNB1* (Figure 9B), *MCM2* (Figure 9C and 9D), *PLK1* (Figure 9E and 9F) and *AURKB* (Figure 9G and 9H) were also increased in patients with metastatic prostate tumors compared to those in patients with primary prostate tumors. These results indicate that those hub genes might be the key downstream genes for PRKAR2B to regulate the development of CRPC.

## DISCUSSION

Castration-resistant prostate cancer (CRPC) is a lethal form of prostate cancer that arises when tumors develop resistance to hormonal therapies. Genomic aberrations drive the acquisition of castration-resistant in CRPC. By analysis of GEO and other databases, we identified that PRKAR2B was anovel gene over-expressing in CRPC and showed that it promotedcell proliferation, invasion and survival. The whole genome transcriptomic analysis of the knock-down of PRKAR2B in CRPC cells showed that PRKAR2B mainly modulates cell cycle gene expression in CRPC, such as *CCNB1*, *MCM2*, *PLK1* and *AURKB*. Our study firstly revealed the undefined function of PRKAR2B in CRPC and provided a promising target gene for further study in treatment of CRPC.

cAMP is an important molecule for a variety of cellular functions. cAMP exerts its effects by activating the cAMP-dependent protein kinase (PKA), which transduces the signal through phosphorylation of different target proteins. PKA is a holoenzyme that is formed by 2 regulatory subunits, type I (R1A and R1B) and type II (R2A and R2B), and 4 catalytic subunits (Cα, Cβ, Cγ, and Cx). The PKA R1A and R2B proteins are the most abundant regulatory subunits of PKA in endocrine tissues.PRKAR2B is the gene encoding PKA type II-beta regulatory subunit(R2B). In adrenocortical human cell line, inactivation of PRKAR2B enhanced cell proliferation and reduced apoptosis [16]. Activation of PKAR2B by selective cAMP analogs alters the growth of adrenocortical cells [17]. A dramatic decrease in PKAR2B protein levels is observed in a subset of adrenocortical adenomas, however, its function is not clear [18]. In contrast to the tumor suppressive function in adrenocortical cells, PKAR2B expression is up-regulated in hormone refractory prostate cancer patients as well as in metastasized prostate tumors [7, 19, 20]. Here, we showed that knockdown of PRKAR2B in CRPC cell line impaired cell survival and invasion, the topaffected biological process is cell cycle progression and also identified that *CCNB1*, *MCM2*, *PLK1* and *AURKB* were down-regulated by knock-down of PRKAR2B. Those genes are all critical elements for cell proliferation and mitosis. CyclinB 1 encoded by CCNB1 is a regulatory protein involved in mitosis. Silencing of cyclin B1 in tumor cells increases susceptibility to taxol and leads to growth arrest *in vivo* [21]. MCM2 belongs to the minichromosome maintenance protein family and plays a major role in DNA replication during the G1-phase of the cell cycle. MCM2 can serve as a cell proliferation marker to differentiate normal and cancer cells [22, 23]. PLK1 is implicated in the dynamic function of the mitotic spindle during chromosome segregation, supporting the functional maturation of the centrosome in late G2/early prophaseand establishment of the bipolar spindle [24, 25]. PLK1 activates Cdc2/cyclin B complex via phosphorylation and activation of Cdc25C and is an early trigger for G2/M transition [26]. Cancer cells depends on PLK1 for survival more than normal cells [27]. Aurora kinases B encoded by AURKB is associate with microtubules during chromosome movement and segregation [28]. Abnormally elevated levels of Aurora B kinase in cancerous cells cause unequal chromosomal separation resulting in formation of cells with abnormal numbers of chromosomes [29]. Regulation of those critical genes of cell cycle progression indicates the important function of PRKAR2B in CRPC.

Although we identified PRKAR2B from CRPC mouse models, and verified its expression in online CRPC patient databases and investigated its function in CRPC cells, some following studies should be performed in the future, such as *in vivo* functional studies and the large scale examination of its expression in CRPC patients.

In summary, we identified that PRKAR2B over expressed in CRPC mouse models and patients, and showed it promoted CRPC cell proliferation, invasion and survival by mainly modulates cell cycle gene expression. Therefore, we conclude that PRKAR2B is a novel oncogenic gene in CRPC, which sheds light on elucidating PRKAR2B function in CRPC and suggest PRKAR2B to be a promising target for future study and treatment of CRPC.

## MATERIALS AND METHODS

### Cell lines and reagents

PC-3,DU-145 and 22RV1 are androgen-independent (castration-resistant) human prostate cancer (Pca) cell lines derived from metastatic sites. LNCaP are androgen-dependent (castration-sensitive) human prostate cancer (Pca) cell line. All the cells are cultured in RPMI1640 supplemented with 10% fetal bovine serum (FBS), 2 mmol/l glutamine, 100units/mL penicillin and 100 μg/mL streptomycin and cultured in a humidified atmosphere of 95% air and 5% CO2 at 37°C.

Antibody directed against totalPRKAR2B was purchased from Thermofisher (Cat. PA5-13799), and antibody directed against Cleaved PARP1 (Cat. 9541S) was purchased from Cell signaling, and Antibody directed against Beta-Actinwas purchased from Sigma (Cat. A2228)

### siRNA interference

PRKAR2BSMART pool (Cat. L-009152-00-0005) was purchased from Dharmacon/Thermo Fisher Scientific. Transfection of the siRNA oligonucleotide duplexes was performed in a 6-well plate (1 × 10^5^cells per well) with Lipofectamine 2000 (Invitrogen, Inc.), using the methods recommended by the manufacturer. Knockdown of PRKAR2B with siRNA was examined after siRNA transfection through western blotting and real-time PCR.

### MTT

Cells were cultured in 24-well plates with 0.5 ml medium per well at 37^°^C. After transient transfection of the cells with PRKAR2B siRNA SMART pool or control siRNA for 24 h,48 h, 72 h and 96 h, the cells were added 50 μL/well of 10 mg/mL 3-(4,5-dimethyl-thiazol-2-yl)-2,5-diphenyltetrazolium bromide (MTT) and incubated for an additional 2 h at 37^°^C. The supernatant was aspirated, and the MTT formazan crystals formed by the cells were dissolved in 500 ul of DMSO. The absorbance was measured at a wavelength of 570nmby a microplate reader [30].

### Western blot

Cells were lysed in a lysis buffer containing 50 mmol/L TRIS-HCl, pH7.4, 150 mmol/L NaCl, 0.5% NP40, 50 mmol/L NaF, 1 mmol/L Na3VO4, 1 mmol/L phenyl-methylsulfonyl fluoride, 25 μg/mL leupeptin, and 25 μg/Ml aprotinin and clarified by centrifugation (14,000 g for 30 min at 4°C). The protein concentration of the cell lysates was determined using the Bradford Coomassie blue method (Pierce Chemical Corp.). Whole-cell lysates were separated by sodium dodecyl sulfate (SDS)-PAGE and transferred onto nitrocellulose membrane. The membranes were blocked with PBS containing 5% (w/v) skim milk at 4°C for 2 h, washed with PBST(PBS with 0.05% Tween-20), and then incubated overnight with primary antibody. After washed with PBST, the membrane was incubated with second antibody at room temperature for 2 h, washed with PBST and then developed with the ECL system. The results of Western blot were analyzed with Odyssey software version 3.0.

### RNA sequencing and differentially expressed genes (DEGs) analysis

DU-145 cell lines with or without PRKAR2B knockdown were subjected to RNA sequencing. RNA sequencing tags were mapped to the human (Homo sapiens) genome (version hg19) using TopHat [31], then the expression abundance (FPKM) value of each gene was estimated by running cufflinks [32] and the differential expressed genes were assessed by cuffdiff. Statistically differentially expressed genes between two groups were those genes with |fold change| > 2 and adjusted *p value* < 0.01. The adjusted *p value* was obtained through applying Benjamini and Hochberg's (BH) false discovery rate correction on the original *p value*, and fold change threshold was selected based on our purpose of focusing on significantly differentially expressed genes.

### Hierarchical clustering

Hierarchical clustering was conducted [33] to classify analyzed samples based on gene expression profiles. Hierarchical clustering using differentially expressed genes (DEGs) demonstrated the global gene expression patterns in the samples. In addition, the DEGs were further extracted and classified in specific biological processes (Gene Ontology terms) and KEGG pathways. The expression pattern of those DEGs was characterized and heat maps of the DEGs were classified in targeted biological processes or KEGG pathways using R package.

### Go and kegg pathway analysis

We utilized R packages–GO.db, KEGG.db and KEGGREST to detect Gene Ontology categories and KEGG pathways with significant enrichment in DEGs comparing to which across all measured genes. The significantly enriched biological processes were identified as *p value* less than threshold value 0.01. As to KEGG pathway, *p value* was set to less than 0.05.

### Real-time qPCR

The mRNA abundances of cell cycle related genes were determined by quantitative real-time PCR assays. The ΔΔCt method of relative quantification and SYBR Green chemistry were used, and β -actin was used as an endogenous control for normalization.

PCR primer sets were designed using Primer Premier 5, and the sequences were as follows: PRKAR2B, 5- TT CGGCGAACTGGCCTTAATG -3 (forward) and 5- ACT TTCAGGCGTTCAGAAAACT -3 (reverse); MCM2, 5 - A TGGCGGAATCATCGGAATCC -3 (forward) and 5 -GGT GAGGGCATCAGTACGC -3 (reverse); AURKB, 5 - CAG TGGGACACCCGACATC-3 (forward) and 5 - GTACACG TTTCCAAACTTGCC -3 (reverse); PLK1, 5 -AAAGAG ATCCCGGAGGTCCTA-3 (forward) and 5 -GGCTGCG GTGAATGGATATTTC-3 (reverse); CCNB1, 5 - AATAAG GCGAAGATCAACATGGC -3 (forward) and 5 - TTTGTT ACCAATGTCCCCAAGAG -3 (reverse);β-actin, 5 - CGTC ATACTCCTGCTTGCTG -3 (forward) and 5 - GTACG CCAACACAGTGCTG.-3 (reverse).

### Cell invasion assay

Cell invasion potential was measured with a Boyden transwell chamber consisting of upper inserts with 8-μm-pore-size filter membranes at the bottom of the inserts and lower wells in 24-well cell culture plates (Corning Life Sciences). Add 20 μl of 1:6 diluted Matrigel (2–3 mg/ml protein) to the center of each cell well inserts. Place coated inserts in incubator to allow the Matrigel to solidify for 20–30 min. Cells (3.5 × 10^5^ cells in 0.2 mL) suspended in serum-free medium with 0.1% bovine serum albumin were seeded into the inserts of the chambers. The inserts were then placed over the wells filled with 0.5 mL 10% FBS culture medium and incubated in a 37°C incubator for 16 h. Cells that had not penetrated the filter membrane in the inserts were wiped off with cotton swabs, and the cells on the underside of the filter membrane were fixed and stained with the HEMA-3 kit (Fisher Diagnostics). Invaded cells were counted in total 10 fields for each sample under microscope with 10X objective and stained cell number per field was calculated [34].

## SUPPLEMENTARY MATERIALS FIGURE


