# Supplementary material for: PRKAR2B plays an oncogenic role in the castration-resistant prostate cancer
Source: Oncotarget. 2016 Dec 20;8(4):6114–29. doi: 10.18632/oncotarget.14044 (PMC5351617; doi:10.18632/oncotarget.14044)
Supplement: Supplementary file 1 [file oncotarget-08-6114-s001.pdf]

PRKAR2B plays an oncogenic role in the castration-resistant prostate cancer

Supplementary Materials

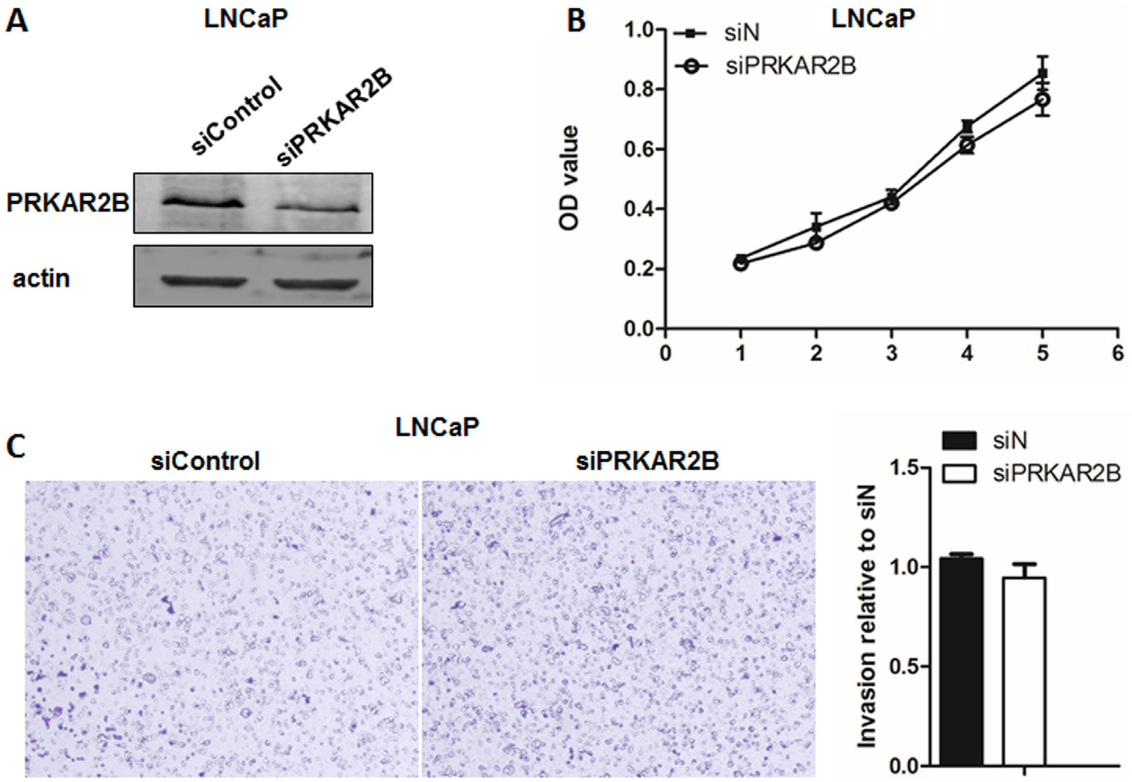

Supplementary Figure S1: Knock-down of *PRKAR2B* expression through siRNA in LNCap cells does not significantly reduce cell growth and invasion. (**A**) Western blot was performed to examine the protein expression level of PRKAR2B in LNCap cells afterknock-down of PRKAR2B using siRNA. Actin is the internal loading control. (**B**) MTT assay showed cell vability in LNCap cells after knock-down of PRKAR2B. OD value was tested at 0 h, 24 h, 48 h, 72 h and 96 h of siRNA transfection. siN: negative control siRNA; siPRKAR2B: PRKAR2B siRNA. (**C**) Cell invasion ability of LNCap cells was examined by invasion chamber assay after knock-down of PRKAR2B for 48 hours. The bar graph shows the average number of the invaded cells per field.
